# Immediate Effects of a Telerehabilitation Program Based on Aerobic Exercise in Women with Fibromyalgia

**DOI:** 10.3390/ijerph18042075

**Published:** 2021-02-20

**Authors:** Ignacio Hernando-Garijo, Luis Ceballos-Laita, María Teresa Mingo-Gómez, Ricardo Medrano-de-la-Fuente, Elena Estébanez-de-Miguel, María Natividad Martínez-Pérez, Sandra Jiménez-del-Barrio

**Affiliations:** 1 Department of Surgery, Ophthalmology and Physiotherapy, Faculty of Health Sciences, University of Valladolid, 42004 Soria, Spain; ignacio.hernando@uva.es (I.H.-G.); luis.ceballos@uva.es (L.C.-L.); tmingo@cir.uva.es (M.T.M.-G.); medranodelafuentericardo@gmail.com (R.M.-d.-l.-F.); 2 Department of Physiatrist and Nursery, Faculty of Health Science, University of Zaragoza, 50009 Zaragoza, Spain; elesteba@unizar.es; 3 Physical and Rehabilitation Department, Santa Barbara Clinical Hospital, 42004 Soria, Spain; mmartinezper@saludcastillayleon.es

**Keywords:** fibromyalgia, chronic pain, exercise, rehabilitation, telerehabilitation

## Abstract

Background: We analyzed the immediate effects of a Telerehabilitation Program (TP) based on aerobic exercise in women with fibromyalgia (FM) syndrome during the lockdown declared in Spain due to the COVID-19 pandemic. Methods: A single-blind randomized controlled trial was designed. Thirty-four women with FM were randomized into two groups: TP group and Control group. The intervention lasted 15 weeks, with 2 sessions per week. The TP based on aerobic exercise was guided by video and the intensity of each session was monitored using the Borg scale. Pain intensity (Visual Analogue Scale), mechanical pain sensitivity (algometer), number of tender points, FM impact (Revised Fibromyalgia Impact Questionnaire), pain catastrophizing (Pain Catastrophizing Scale), physiological distress (Hospital Anxiety and Depression Scale), upper (Arm Curl Test) and lower-limb physical function (6-min Walk Test) were measured at baseline and after the intervention. Results: The TP group improved pain intensity (*p* = 0.022), mechanical pain sensitivity (*p* < 0.05), and psychological distress (*p* = 0.005), compared to the Control group. The Control group showed no statistically significant changes in any variable (*p* > 0.05). Conclusions: A TP based on aerobic exercise achieved improvements on pain intensity, mechanical pain sensitivity, and psychological distress compared to a Control group during the lockdown declared in Spain due to COVID-19 pandemic.

## 1. Introduction

Fibromyalgia (FM) is a chronic disorder characterized by persistent widespread pain and commonly associated with psychological distress, pain catastrophizing and lower levels of physical function among other symptoms [1,2,3]. The FM worldwide prevalence has been estimated in 2.1% and has shown to be higher among women, at a rate of 4:1 [4].

The World Health Organization declared in March 2020 the COVID-19 as a global pandemic [5]. Most countries carried out measures such as lockdown and restrictions to prevent the spread of COVID-19 and reduce the infection risk. These restrictions have resulted in negative effects by limiting the participation in normal daily living, social and physical activities, and the access to many forms of exercise [6,7]. The direct and indirect consequences derived from the pandemic situation have affected the health status of patients with chronic diseases [8]. Patients with FM have reported an increase in pain intensity, pain catastrophizing, physiological distress, and sedentary lifestyles affecting negatively to the quality of life and social aspect of these patients [3,9,10,11].

Clinical guidelines recommend the use of conservative non-pharmacological therapies as a first-line intervention for patients with FM [12,13]. The aerobic exercise is one of the most accepted modalities. Recent studies showed that individualized low-intensity aerobic exercise was well-tolerated by patients with FM [14,15,16]. These studies achieved positive effects on pain intensity, FM impact, hyperalgesia, physical function, catastrophizing, and psychological distress [14,15,16], without major adverse events [14]. In addition, aerobic exercise showed lower costs and larger positive effects compared to usual care [17].

Given the high professional and economic requirements in the health care system during the COVID-19 pandemic, the design of therapeutic strategies without the risk of viral infection has become necessary, and telerehabilitation has been proposed as a feasible method [18]. Telerehabilitation has been developed in the last years and implies the substitution of traditional face-to-face approaches by telematic procedures, using telephone, video, and computer technologies [19]. Telerehabilitation allows guide some therapies to maintain social distance, while keeping the therapist–patient communication and monitoring [20,21,22].

Our hypothesis is that a telerehabilitation program (TP) based on aerobic exercise could improve the main FM symptoms in patients with FM.

Therefore, the purpose of this study was to analyze the immediate effects on pain intensity, mechanical pain sensitivity, FM impact, pain catastrophizing, psychological distress, and physical function of a TP based on aerobic exercise in patients with FM during the period of mobility restrictions imposed by the COVID-19 pandemic.

## 2. Material and Methods

### 2.1. Study Design

We conducted a single-blind randomized controlled trial to analyze the effects of a TP based on aerobic exercise in patients with FM. This study was carried out at the Faculty of Health Sciences of the University of Valladolid from March to June 2020 (lockdown period in Spain) and was developed according to CONSORT guidelines. The ethical approval was obtained from the Research Ethics Committee of Valladolid Este (19-410) and the study was registered at clinicaltrials.gov (NCT04340674).

### 2.2. Participants

The participants were referred by a rheumatologist from February to March 2020. Thirty-seven patients diagnosed with FM (mean years since diagnosis ± SD: 10.54 ± 7.4) were screened of which 37 women aged from 30 to 75 (mean ± SD: 53.44 ± 8.8) years, participated in the study.

The inclusion criteria were: women diagnosed with FM by a rheumatologist according to the latest diagnostic criteria of the American College of Rheumatology [23], aged from 30 to 75 years, and having access to internet with any type of digital device.

The exclusion criteria were: presence of other systemic, somatic, or psychiatric diseases, pregnancy or lactation, previous physiotherapy treatments, or modifications in pharmacological treatments during the study or in the last 3 months before the intervention, exercise contraindications, or inability to communicate with examiners.

### 2.3. Sample Size

The calculation of the sample size was performed based on the primary dependent variable (pain intensity). Seventeen patients per group were estimated assuming a standard deviation of 1.9 previously reported in a pilot study in patients with FM and a between-group mean difference of 2 cm in the Visual Analogue Scale (VAS), which is considered as the minimum relevant change, establishing an α error of 5%, a β error of 20%, and a follow-up loss rate of 15%. The software used for the sample size calculation was Minitab^®^ program version 13.0.

### 2.4. Randomization

All the participants that met the inclusion and exclusion criteria were randomly allocated to the TP group or to the Control group. The allocation (ratio 1:1) was performed by an independent researcher who did not participate in the data acquisition or statistical analysis, using the GraphPad computer software 2018 (GraphPad Software, San Diego, USA) [24].

### 2.5. Interventions

The TP group received 15 weeks of a telerehabilitation aerobic exercise program. The Control group received no additional interventions. Both the TP group and the Control group were asked to maintain the same medical prescription during the study.

The intervention was performed by an experienced physiotherapist with more than 4 years of clinical experience in therapeutic exercise for chronic diseases, who was blinded to the measurements.

The TP group completed 30 sessions of exercise, during 15 weeks, with 2 sessions per week [20,25]. Telerehabilitation sessions were based on low-impact rhythmic movements, guided by video, according to the protocol of Schachter et al. [26]. The TP was designed according to American Pain Society guidelines on FM, that recommended moderately intense aerobic exercise 2–3 times per week [27] and included 3 parts: warm-up, central part, and cool-down. Each session lasted 50 min.

The warm-up consisted of joint mobility exercises and active stretching. In the central part, the aerobic exercises were based on low-impact rhythmic movements guided by video. The repetition rate determines the intensity of the exercises: 10, 15, 20, or 25 repetitions per minute. The patients could watch the four intensities of each exercise performed by the therapist on video. The first two sessions were supervised before telerehabilitation sessions to ensure that the patients performed the exercises in a correct and safe way and allowed to establish the intensity of the protocol at the beginning of the intervention. The exercise intensity of each telerehabilitation session was individually adjusted by the participants’ Modified Borg perceived exertion reported in previous session. The Modified Borg Scale ranges from 0 to 10 points with higher scores representing higher physical exertion [28] and has shown to be a valid tool to determine intensity in FM patients [29]. The exercise intensity was increased if the Borg rate was less than 4 and intensity was decreased if the Borg rate was more than 7, according to the protocol described by Duruturk et al. [30] The cool-down part was based on statis stretching of the major muscles (3 sets of 30 s) and breathing techniques. The physiotherapist, who performed the intervention, adapted and controlled all the exercises to avoid any adverse event and to ensure patients’ safety.

Participants of both groups were called by video call, once a week. The participants of the TP group were called to control and individualize the exercises. The participants of the Control group were contacted to ensure that they maintained the same conditions during the study.

The treatment adherence was registered using an online attendance record Google Forms software (Google LLC, Menlo Park, USA).

### 2.6. Outcome Measures

The measurements were performed the previous week before the lockdown, by two physiotherapists blinded to the group assignment. Clinical and demographic information including age, sex, height, weight, body mass index, and medication intake were recorded at baseline. All the outcome measures were assessed at baseline and after the intervention.

Primary outcome

The main outcome measure was pain intensity. Pain intensity was registered using the 10-cm VAS, in which 0 represented “no pain” and 10 represented “the most intense pain imaginable.” This scale showed excellent reliability in chronic pain subjects, with an Intraclass Correlation Coefficient (ICC) of 0.97–0.99 [31,32].

Secondary outcomes

Mechanical pain sensitivity was evaluated with an analogical pressure algometer (Psymtec, FPK 20, Wagner Instruments, Greenwich, USA). The 18 tender points described in the 1990 ACR criteria were assessed [33]. The tender points were right and left occiput, low cervical, trapezius, supraspinatus, second rib, lateral epicondyle, gluteal, greater trochanter, and knee. The mechanical pain sensitivity threshold was measured with the algometer applying increasing pressure on each tender point where the sense of pressure change to pain was registered as the pain threshold. Pressure was increased at a rate of 1 kg/s. Participants were instructed to say “stop” when the pressure became to clear sensation of pain. The number of tender points and the algometer score were analyzed after the measurements. A tender point was considered if the threshold was ≤ 4 kg/cm^2^ [33]. The algometer score was calculated as the sum of the pain-pressure values obtained for each tender point. Mechanical pain sensitivity measured through algometry has shown good to excellent reliability in different body regions (ICC = 0.82–0.97) [34,35].

FM impact was measured using the Spanish version of the Revised Fibromyalgia Impact Questionnaire (FIQ-R). This questionnaire consists of 21 questions based on an 11-point numeric rating scale of 0 to 10. The scores range from 0 to 100, with higher scores representing higher FM impact. The reliability of the questionnaire has shown to be excellent (ICC = 0.81) [36].

The Spanish version of the Pain Catastrophizing Scale (PCS) was used to measure catastrophizing cognitions related to pain. This scale assesses the dimensions of rumination, magnification, and helplessness and consists of 14 items, with each item rated from 0 (not at all) to 4 (all the time). The total score ranged from 0 to 52 points, and higher values show higher pain catastrophizing. Total score and dimensions were registered. The Spanish version of PCS has shown good test–retest reliability in FM patients (ICC = 0.84) [37].

Psychological distress was measured using the Hospital Anxiety and Depression Scale (HADS). The scale includes an anxiety subscale (HADS-A) and a depression subscale (HADS-D), with 7 items into each subscale. Subscale scores range from 0 to 21, with higher scores representing higher anxiety or depression levels. Total score and subscales were registered. The HADS has shown good test–retest reliability (ICC = 0.84–0.94) [38,39].

The upper and lower limbs’ physical function was measured with the 6-min Walk Test (6MWT) and with the Arm Curl Test (ACT), respectively. The 6MWT measures the maximum distance in meters that an individual is able to walk, as fast as possible, during the period of 6 min. In the ACT test, the patients, seated on a chair with backrest and holding a dumbbell (2.3 kg) with the elbow extended, had to flex and extend the elbow as many times as possible in 30 s [40]. Both tests have shown excellent reliability in FM patients (6MWT ICC: 0.91–0.98; ACT ICC: 0.81–0.86) [41,42].

### 2.7. Statistical Analysis

An assessor blinded to the treatment allocation conducted the statistical analysis using SPSS software, version 24.0. The statistical analysis was conducted according to intention-to-treat (ITT). Mean and standard deviations were calculated for quantitative variables. Frequencies and percentages were calculated for qualitative variables. The Shapiro–Wilk test was used to assess the normal distribution of quantitative variables. Between-group comparisons of clinical and demographic variables were analyzed using the Student´s *t*-test for normally distributed data or the Mann–Whitney U test for non-normally distributed data. Chi-square test (X^2^) was used for between-group comparison of nominal variables.

A two-way ANOVA was used to analyze the differences in outcomes with time (baseline and postintervention) as the within-subjects factor and group (TP group and Control group). A *p*-value < 0.05 was considered statistically significant. The effect size (Cohen’s d) was also calculated, to estimate the magnitude of the differences within and between groups. The magnitude of difference was classified as small if the value of Cohen’s d ranged from 0.2 to 0.5, as moderate if it ranged from 0.5 to 0.8 or, as large if Cohen’s d value was greater than 0.8.

## 3. Results

**Figure 1:**
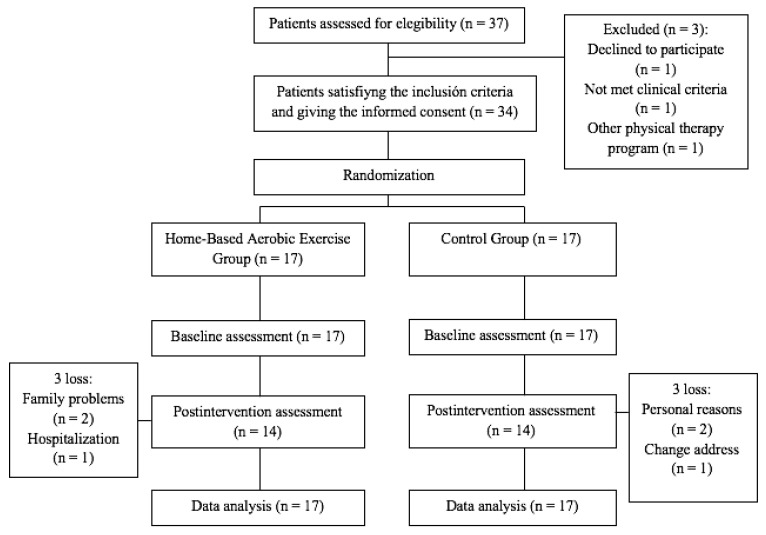
Flowchart diagram.

Thirty-seven patients diagnosed with FM were screened. Three patients were excluded: 2 patients did not meet the inclusion criteria and 1 patient declined to participate for personal reasons. Thirty-four female patients that met the eligibility criteria signed the informed consent and were randomized into the TP group (*n* = 17) or the Control group (*n* = 17). Six patients failed to complete the study: three patients in the TP group and three patients in the Control group. The flowchart diagram of the recruitment and follow-up of participants is shown in Figure 1. Demographic and medication intake at baseline are shown in Table 1. No statistically significant differences were found between both groups at baseline (*p* > 0.05). Adherence to the TP group over the 10 weeks was high, averaging 89.9%.

After the intervention, a two-way ANOVA showed significant Group by Time interactions for pain intensity (F = 5.99; *p* = 0.021), mechanical pain sensitivity (Algometer score: F = 10.67; *p* = 0.003; active points: F = 7.90; *p* = 0.009), and psychological distress (F = 12.03; *p* = 0.002). The TP group showed a greater decrease in pain intensity (Δ1.53 (0.04 to 3.03) and in psychological distress (Δ9.54 (3.09 to 15.99) and a greater increase in mechanical pain sensitivity (algometer score: Δ−14.06 (−26.20 to −1.92); tender points: Δ2.65 (0.18 to 5.11) than the Control group. The results achieved for these variables in the PT group showed a large effect size (>0.8). There were no statistically significant differences between both groups on FM impact, pain catastrophizing, and physical function (*p* > 0.05).

Regarding within-group change scores, the PT group showed a statistically significant improvement in all the variables (*p* < 0.05) except in the rumination PCS subscale (*p* = 0.078) and in the 6MWT (*p* = 0.051). No within-groups differences were found in any variable in the Control group (*p* > 0.05). Table 2 provides before and after intervention data, within- and between-groups differences as well as the effect size for all the dependent variables.

## 4. Discussion

This randomized controlled clinical trial is the first study to investigate the effects of a TP based on aerobic exercise in women with FM during the lockdown caused by COVID-19 pandemic. The results reported in this study showed that the TP group achieved statistically significant improvements on pain intensity, mechanical pain sensitivity, and psychological distress compared to a Control group.

Pain intensity, mechanical pain sensitivity, and psychological distress improved after TP intervention. The change achieved on pain intensity was superior to the MCID (Minimal Clinically Important Difference) described for patients with chronic pain [43]. The change achieved on psychological distress was statistically significant and superior to the MCID stated for patients with chronic disease [44]. However, the mean value at the end of the treatment was higher than 8 points, considered the marked score for the diagnosis of psychological distress [45]. These results are similar to previous studies showing that aerobic exercise reduces pain intensity, mechanical pain sensitivity, and psychological distress [16,46,47,48,49], however, in this clinical trial, we achieved improvements with a TP without face-to-face sessions.

Regular exercise has shown to modify the levels of neurotransmitters, neuromodulators, and the hypothalamic–pituitary function [50,51]. The changes in these elements are related to improvements on pain, stress, anxiety, and depression among others in patients with chronic pain [52,53]. In this way, the analgesic effect of exercise has shown to be related to higher levels of endorphins [54]. Thus, the improvements on pain, mechanical pain sensitivity, and psychological distress achieved in our study could be related to the increase in neurotransmitters’ levels, especially, endorphins, release by the hypothalamus [50,51,54].

The TP group showed improvements on FM impact, pain catastrophizing, and upper limb physical function. However, these changes were not enough to report statistically significant differences between groups. The results of this study are in accordance with previous studies that found no differences comparing therapeutic exercise to usual care [25,55]. The intervention applied to the TP group could be an insufficient stimulus to improve these variables. Concerning pain catastrophizing and FM impact, the aerobic exercise in isolation could not be able to modify the beliefs and cognitions about pain. Other studies reported that the addition of pain neurophysiology education to exercise therapy seems to report better benefits on pain catastrophizing and FM impact than exercise therapy in isolation [56,57,58].

Regarding the physical function, only the TP group showed a statistically significant improvement in the upper limb physical function. No within- or between-groups differences were found for the rest of physical function variables. A recent systematic review reported that aerobic exercise improves physical function [14], however, it is important to mention that the current clinical trial was performed during the lockdown situation, so the patients were not allowed to go out. The social and mobility restrictions during the study period may explain the lack of improvements achieved in these variables.

From a clinical perspective, this study provides evidence suggesting that a TP intervention based on aerobic exercise could be an effective strategy in FM patients, in the exceptional situation caused by the COVID-19 pandemic. TP intervention could improve FM symptoms in a safe way, without adverse events, and show high adherence level. The individualized supervision performed by the physiotherapist allowed monitoring the type of exercises, the intensity of the sessions, and the status of the patients, which seems to be necessary to achieve a high adherence level and healthy habits in patients with FM.

The present study has potential limitations. First, men were not included in this study, so results cannot be generalized to the entire population. Second, the intervention was not compared with other experimental interventions, such a supervised exercise program. Third, the reduced small sample size considered in the study made it difficult to achieve between-group changes in some variables. Finally, there was a lack of follow-up analysis, for this reason, the long-term effects of the study intervention were not reported.

## 5. Conclusions

The results of this study showed that a TP based on aerobic exercise was effective for reducing pain intensity, mechanical pain sensitivity, and psychological distress during the lockdown caused by COVID-19 pandemic.

## Figures and Tables

**Table 1:** Baseline characteristics of the study population.

	TP Group Mean ( ± SD)	Control Group Mean ( ± SD)	Significance
Age (years)	51.81 ± 9.05	55.06 ± 8.51	0.304 ^a^
Height (cm)	158.63 ± 6.29	161.81 ± 5.13	0.191 ^b^
Weight (kg)	68.19 ± 16.88	68.13 ± 15.10	0.836 ^b^
BMI (kg/cm^2^)	27.25 ± 7.30	25.93 ± 5.27	0.598 ^b^
Medication *n* (%)	17 (100)	17 (100)	0.224 ^c^
Anxiolytics *n* (%)	9 (53)	7 (41)	
Antidepressants *n* (%)	9 (53)	9 (53)	
Anti-inflammatory *n* (%)	14 (82)	8 (47)	
Analgesic *n* (%)	11 (65)	12 (71)	
Muscle relaxant *n* (%)	4 (24)	2 (12)	

^a^: Student´s *t*-test; ^b^: Mann–Whitney U; ^c^: Chi-square. Values for quantitative data are expressed in mean and standard deviation, and values for medication are expressed as percentages (%) and frequencies (*n*).

**Table 2:** Primary and secondary outcomes at baseline and after intervention as well as within-group mean scores.

	Baseline Mean ± SD (95% CI)	Postintervention Mean ± SD (95% CI)	Within-Group Changes *p*-Values	Within-Group Effect Sizes (Cohen’s d)	Between-Groups *p*-Value	Between-Groups Effect Sizes (Cohen’s d)
VAS (0–10)						
TP group	7.08 ± 1.45 (6.38 to 7.78)	4.92 ± 2.00 (3.85 to 6.00)	2.15 (1.37, 2.94) <0.001	−1.2	F = 5.99 0.021	0.8
Control group	7.29 ± 1.07 (6.62 to 7.96)	6.46 ± 1.92 (5.42 to 7.50)	0.82 (−0.3, 1.68) 0.058	−0.5		
Algometer score (kg/cm^2^)						
TP group	45.42 ± 12.56 (37.92 to 52.92)	56.85 ± 15.28 (48.43 to 65.28)	−11.43 (−18.38, −4.48) 0.004	0.8	F = 10.67 0.003	0.9
Control group	45.20 ± 14.68 (37.49 to 52.98)	42.79 ± 15.32 (34.05 to 51.53)	2.40 (−3.48, 8.29) 0.391	−0.1		
Tender points						
TP group	16.00 ± 3.38 (14.42 to 17.57)	14.13 ± 4.12 (12.42 to 15.84)	1.86 (0.56, 3.17) 0.008	−0.5	F = 7.90 0.009	0.8
Control group	16.50 ± 2.47 (14.86 to 18.13)	16.78 ± 1.84 (15.01 to 18.55)	−0.28 (−1.25,0.68) 0.537	0.1		
FIQ-R						
TP group	59.44 ± 9.04 (52.14 to 66.74)	44.00 ± 15.21 (34.10 to 53.91)	15.43 (7.81, 23.05) 0.001	−1.2	F = 1.36 0.254	0.1
Control group	55.36 ± 16.46 (48.06 to 62.66)	46.90 ± 20.47 (36.99 to 56.81)	8.45 (−1.99, 18.90) 0.104	−0.4		
PCS						
TP group	24.8 ± 12.0 (18.27 to 29.47)	17.6 ± 12.4 (14.19 to 28.17)	7.00 (1.19, 12.80) 0.022	−0.6	F = 0.415 0.525	0.4
Control group	24.10 ± 10.8 (15.72 to 28.40)	23.5 ± 14.0 (13.45 to 27.70)	1.07 (−4.54, 6.68) 0.687	0.0		
Rumination						
TP group	7.2 ± 3.8 (5.32 to 9.53)	5.1 ± 4.7 (2.45 to 7.69)	2.34 (−0.29, 5.01) 0.078	−0.5	F = 1.91 0.169	0.4
Control group	7.2 ± 3.6 (5.15 to 9.09)	6.9 ± 4.9 (4.42 to 9.32)	0.25 (−1.65, 2.15) 0.783	0.0		
Helplessness						
TP group	12.1 ± 6.3 (8.80 to 14.91)	8.9 ± 6.1 (5.19 to 12.65)	2.92 (0.46, 5.39) 0.023	−0.5	F = 0.430 0.518	0.2
Control group	12.4 ± 5.2 (9.07 to 14.79)	10.2 ± 7.4 (6.69 to 13.67)	1.75 (−1.13, 4.63) 0.215	−0.3		
Magnification						
TP group	5.5 ± 2.9 (3.81 to 6.75)	3.6 ± 2.4 (2.97 to 5.07)	1.71 (0.38, 3.04) 0.015	−0.7	F = 2.05 0.163	0.1
Control group	4.4 ± 2.5 (3.12 to 5.87)	3.9 ± 3.0 (2.53 to 5.34)	0.56 (−0.55, 1.67) 0.300	−0.1		
HADS						
TP group	20.52 ± 6.83 (16.76 to 24.29)	11.70 ± 8.74 (7.21 to 16.19)	8.82 (3.33, 14.30) 0.004	−1.1	F=12.03 0.002	1.0
Control group	20.43 ± 8.37 (16.55 to 24.32)	21.25 ± 9.42 (16.62 to 25.88)	−0.81 (−2.50, 0.87) 0.321	0.0		
HADS-A						
TP group	10.88 ± 3.38 (9.02 to 12.73)	6.29 ± 5.13 (3.86 to 8.72)	4.58 (1.69, 7.47) 0.004	−1.0	F = 10.25 0.003	0.9
Control group	10.81 ± 4.10 (8.90 to 12.72)	11.00 ± 4.66 (8.49 to 13.05)	−0.18 (−1.27, 0.89) 0.718	0.0		
HADS-D						
TP group	9.64 ± 4.16 (7.49 to 11.81)	5.41 ± 4.13 (3.09 to 7.72)	4.23 (1.51, 6.95) 0.005	−1.0	F = 2.97 0.001	1.0
Control group	9.62 ± 4.58 (7.34 to 11.85)	10.31 ± 5.19 (7.92 to 12.69)	−0.68 (−1.65, 0.27) 0.151	0.1		
6MWT						
TP group	403.57 ± 107.13 (327.90 to 480.14)	434.72 ± 73.78 (367.94 to 501.50)	−30.70 (14.13, −61.50) 0.051	0.3	F = 1.54 0.225	−0.2
Control group	407.01 ± 137.09 (326.60 to 473.35)	411.72 ± 145.83 (347.37 to 476.07)	−11.71 (−26.16, 2.73) 0.104	0.0		
ACT						
TP group	9.60 ± 4.31 (6.28 to 12.01)	11.38 ± 4.27 (8.57 to 14.19)	−2.23 (−3.65, −0.81) 0.005	0.4	F = 3.98 0.056	−0.2
Control group	10.06 ± 5.56 (7.39 to 12.73)	10.33 ± 5.42 (7.72 to 12.94)	−0.26 (−1.81, 1.27) 0.717	0.0		

VAS: Visual Analogue Scale; FIQ-R: Fibromyalgia Impact Questionnaire; PCS: Pain Catastrophizing Scale; HADS: Hospital Anxiety and Depression Scale; HADS-A: Hospital Anxiety and Depression Scale–Anxiety Subscale: HADS-D: Hospital Anxiety and Depression Scale–Depression Subscale; 6MWT: 6-min Walk Test; ACT: Arm Curl Test.

## Data Availability

Data sharing not applicable.

## References

[1] Sarzi-Puttini P., Atzeni F., Salaffi F., Cazzola M., Benucci M., Mease P.J. (2011). Multidisciplinary approach to fibromyalgia: What is the teaching?. Best Pract. Res. Clin. Rheumatol..

[2] Chinn S., Caldwell W., Gritsenko K. (2016). Fibromyalgia pathogenesis and treatment options update. Curr. Pain Headache Rep..

[3] Borchers A.T., Gershwin M.E. (2015). Fibromyalgia: A critical and comprehensive review. Clin. Rev. Allergy Immunol..

[4] Cabo-Meseguer A., Cerdá-Olmedo G., Trillo-Mata J.L. (2017). Fibromyalgia: Prevalence, epidemiologic profiles and economic costs. Med. Clín. (Engl. Ed.).

[5] World Health Organization (2020). WHO Director-General’s Opening Remarks at the Media Briefing on COVID-19. https://www.who.int/dg/speeches/detail/who-director-general-s-opening-remarks-at-the-media-briefing-on-covid-19,7-September-2020.

[6] Bloch W., Halle M., Steinacker J.M. (2020). Sport in times of corona. Dtsch Z Sportmed..

[7] Hossain M.M., Sultana A., Purohit N. (2020). Mental health outcomes of quarantine and isolation for infection prevention: A systematic umbrella review of the global evidence. SSRN Electron J..

[8] Pope J.E. (2020). What does the COVID-19 pandemic mean for rheumatology patients?. Curr. Treat. Options Rheumatol..

[9] Ping W., Zheng J., Niu X., Guo C., Zhang J., Yang H., Shi Y. (2020). Evaluation of health-related quality of life using EQ-5D in China during the COVID-19 pandemic. PLoS ONE.

[10] Miyachi M., Winfree K., Woodbury A., Gavilán-Carrera B., Segura-Jiménez V., Acosta-Manzano P., Borges-Cosic M., Álvarez-Gallardo I.C., Delgado-Fernández M. (2020). Patterns of sedentary time and quality of life in women with fibromyalgia: Cross-sectional study from the Al-ándalus project. JMIR mHealth uHealth.

[11] Galvez-Sánchez C.M., Montoro C.I., Duschek S., Del Paso G.A.R. (2020). Pain catastrophizing mediates the negative influence of pain and trait-anxiety on health-related quality of life in fibromyalgia. Qual. Life Res..

[12] De Miquel C.A., Campayo J.G., Flórez M.T., Arguelles J.M.G., Tarrio E.B., Montoya M.G., Pérez Á.M., Martínez A.S., Vidal Fuentes J., Altarriba Alberch E. (2010). Interdisciplinary consensus document for the treatment of fibromyalgia. Actas Esp. Psiquiatr..

[13] Macfarlane G.J., Kronisch C., Dean L.E., Atzeni F., Häuser W., Fluß E., Choy E., Kosek E., Amris K., Branco J. (2016). EULAR revised recommendations for the management of fibromyalgia. Ann. Rheum. Dis..

[14] Bidonde J., Busch A.J., Schachter C.L., Overend T.J., Kim S.Y., Góes S.M., Boden C., Foulds H.J. (2017). Aerobic exercise training for adults with fibromyalgia. Cochrane Database Syst. Rev..

[15] Sosa-Reina M.D., Nunez-Nagy S., Gallego-Izquierdo T., Pecos-Martín D., Monserrat J., Álvarez-Mon M. (2017). Effectiveness of therapeutic exercise in fibromyalgia syndrome: A systematic review and meta-analysis of randomized clinical trials. BioMed Res. Int..

[16] Izquierdo-Alventosa R., Inglés M., Cortés-Amador S., Gimeno-Mallench L., Chirivella-Garrido J., Kropotov J., Serra-Añó P. (2020). Low-intensity physical exercise improves pain catastrophizing and other psychological and physical aspects in women with fibromyalgia: A randomized controlled trial. Int. J. Environ. Res. Public Health.

[17] Miyamoto G.C., Lin C.-W.C., Cabral C.M.N., Van Dongen J.M., Van Tulder M.W. (2018). Cost-effectiveness of exercise therapy in the treatment of non-specific neck pain and low back pain: A systematic review with meta-analysis. Br. J. Sports Med..

[18] Cohen S.P., Baber Z.B., King L.T.C.S., Fowler C.D.R.I.M., Stojanovic M.P., Hayek S.M., Phillips C.D.R.C.R., Buvanendran A., McLean L.T.C.B.C., Chen Y. (2020). Pain management best practices from multispecialty organizations during the COVID-19 pandemic and public health crises. Pain Med..

[19] Peretti A., Amenta F., Tayebati S.K., Nittari G., Mahdi S.S. (2017). Telerehabilitation: Review of the state-of-the-art and areas of application. JMIR Rehabil. Assist. Technol..

[20] Harden R.N., Song S., Fasen M.J., Saltz D.S.L., Nampiaparampil D., Vo A., Revivo D.G. (2012). Home-based aerobic conditioning for management of symptoms of fibromyalgia: A pilot study. Pain Med..

[21] Coultas D.B., Jackson B.E., Russo R., Peoples J., Singh K.P., Sloan J., Uhm M., Ashmore J.A., Blair S.N., Bae S. (2018). Home-based physical activity coaching, physical activity, and health care utilization in chronic obstructive pulmonary disease. Chronic obstructive pulmonary disease self-management activation research trial secondary outcomes. Ann. Am. Thorac. Soc..

[22] Preston E., Dean C.M., Ada L., Stanton R., Brauer S., Kuys S., Waddington G. (2017). Promoting physical activity after stroke via self-management: A feasibility study. Top. Stroke Rehabil..

[23] Wolfe F., Clauw D.J., Fitzcharles M.-A., Goldenberg D.L., Häuser W., Katz R.L., Mease P.J., Russell I.J., Russell A.S., Walitt B. (2016). 2016 revisions to the 2010/2011 fibromyalgia diagnostic criteria. Semin. Arthritis. Rheum..

[24] Suresh K.P. (2011). An overview of randomization techniques: An unbiased assessment of outcome in clinical research. J. Hum. Reprod. Sci..

[25] Da Costa D., Abrahamowicz M., Lowensteyn I., Bernatsky S., Dritsa M., Fitzcharles M.-A., Dobkin P.L. (2005). A randomized clinical trial of an individualized home-based exercise programme for women with fibromyalgia. Rheumatology.

[26] Schachter C.L., Busch A.J., Peloso P.M., Sheppard M.S. (2003). Effects of short versus long bouts of aerobic exercise in sedentary women with fibromyalgia: A randomized controlled trial. Phys. Ther..

[27] Buckhardt C.S., Goldenberg D., Crofford L., Gerwin R., Gowens S., Jackson K., Kugel P., McCarberg W., Rudin N., Schanberg L. (2005). Guideline for the management of fibromyalgia syndrome pain in adults and children. Glenview Am. Pain. Soc..

[28] Borg A.G. (1982). Psychophysical bases of perceived exertion. Med. Sci. Sports Exerc..

[29] Soriano-Maldonado A., Ruiz J.R., Álvarez-Gallardo I.C., Segura-Jiménez V., Santalla A., Munguía-Izquierdo D. (2015). Validity and reliability of rating perceived exertion in women with fibromyalgia: Exertion-pain discrimination. J. Sports Sci..

[30] Duruturk N., Tuzun E.H., Culhaoglu B. (2014). Is balance exercise training as effective as aerobic exercise training in fibromyalgia syndrome?. Rheumatol. Int..

[31] Alfonsin M.M., Chapon R., de Souza C.A., Genro V.K., Mattia M.M., Cunha-Filho J.S. (2019). Correlations among algometry, the visual analogue scale, and the numeric rating scale to assess chronic pelvic pain in women. Eur. J. Obstet. Gynecol. Reprod. Biol. X.

[32] Alghadir A.H., Anwer S., Iqbal A., Iqbal Z.A. (2018). Test–retest reliability, validity, and minimum detectable change of visual analog, numerical rating, and verbal rating scales for measurement of osteoarthritic knee pain. J. Pain Res..

[33] Wolfe F., Smythe H.A., Yunus M.B., Bennett R.M., Bombardier C., Goldenberg D.L., Tugwell P., Campbell S.M., Abeles M., Clark P. (1990). The american college of rheumatology. Criteria for the classification of fibromyalgia. Arthritis Rheum..

[34] Mutlu E.K., Ozdincler A.R. (2015). Reliability and responsiveness of algometry for measuring pressure pain threshold in patients with knee osteoarthritis. J. Phys. Ther. Sci..

[35] Knapstad M.K., Nordahl S.H.G., Naterstad I.F., Ask T., Skouen J.S., Goplen F.K. (2018). Measuring pressure pain threshold in the cervical region of dizzy patients-The reliability of a pressure algometer. Physiother. Res. Int..

[36] Monterde S., Salvat I., Montull S., Fernández-Ballart J. (2004). Validación de la versión española del Fibromyalgia Impact Questionnaire. Rev. Española Reumatol..

[37] García Campayo J., Rodero B., Alda M., Sobradiel N., Montero J., Moreno S. (2008). Validation of the Spanish version of the Pain Catastro-phizing Scale in fibromyalgia. Med. Clin. (Barc.).

[38] Michopoulos I., Douzenis A., Kalkavoura C., Christodoulou C., Michalopoulou P., Kalemi G., Fineti K., Patapis P., Protopapas K., Lykouras L. (2008). Hospital anxiety and depression scale (HADS): Validation in a Greek general hospital sample. Ann. Gen. Psychiatry.

[39] Reda A.A. (2011). Reliability and validity of the ethiopian version of the hospital anxiety and depression scale (Hads) In HIV infected patients. PLoS ONE.

[40] Segura-Jiménez V., Soriano-Maldonado A., Estévez-López F., Álvarez-Gallardo I.C., Delgado-Fernández M., Ruiz J.R., Aparicio V.A. (2016). Independent and joint associations of physical activity and fitness with fibromyalgia symptoms and severity: The al-Ándalus project. J. Sports Sci..

[41] Leon-Llamas J.L., Villafaina S., Murillo-Garcia A., Collado-Mateo D., Domínguez-Muñoz F.J., Sánchez-Gómez J., Gusi N. (2019). Strength as-sessment under dual task conditions in women with fibromyalgia: A test–retest reliability study. Int. J. Environ. Res. Public Health.

[42] Pankoff B.A., Overend T.J., Lucy S.D., White K.P. (2000). Reliability of the six-minute walk test in people with fibromyalgia. Arthritis Rheum..

[43] Tubach F., Ravaud P., Baron G., Falissard B., Logeart I., Bellamy N., Bombardier C., Felson D., Hochberg M., Van Der Heijde D. (2005). Evaluation of clinically relevant changes in patient reported outcomes in knee and hip osteoarthritis: The minimal clinically important improvement. Ann. Rheum. Dis..

[44] Smid D.E., Franssen F.M., Houben-Wilke S., Vanfleteren L.E., Janssen D.J., Wouters E.F., Spruit M.A. (2017). Responsiveness and MCID estimates for cCAT, CCQ, and HADS in patients with COPD undergoing pulmonary rehabilitation: A prospective analysis. J. Am. Med. Dir. Assoc..

[45] Zigmond A.S., Snaith R.P. (1983). The hospital anxiety and depression scale. Acta Psychiatr. Scand..

[46] Andrade C.P., Zamunér A.R., Forti M., Tamburús N.Y., Silva E. (2019). Effects of aquatic training and detraining on women with fibromyalgia: Controlled randomized clinical trial. Eur. J. Phys. Rehabil. Med..

[47] De Assis M.R., Silva L.E., Alves A.M.B., Pessanha A.P., Valim V., Feldman D., Neto T.L.D.B., Natour J. (2006). A randomized controlled trial of deep water running: Clinical effectiveness of aquatic exercise to treat fibromyalgia. Arthritis Rheum..

[48] Fernandes G., Jennings F., Cabral M.V.N., Buosi A.L.P., Natour J. (2016). Swimming improves pain and functional capacity of patients with fibromyalgia: A randomized controlled trial. Arch. Phys. Med. Rehabil..

[49] Gowans S.E., Dehueck A., Voss S., Silaj A., Abbey S.E., Reynolds W.J. (2001). Effect of a randomized, controlled trial of exercise on mood and physical function in individuals with fibromyalgia. Arthritis Rheum..

[50] Barclay T., Richards S., Schoffstall J., Magnuson C., McPhee C., Price J., Aita S., Anderson A., Johnson D., Price J. (2014). A pilot study on the effects of exercise on depression symptoms using levels of neurotransmitters and EEG as markers. Eur. J. Psychol. Educ. Stud..

[51] Lopresti A.L., Hood S.D., Drummond P.D. (2013). A review of lifestyle factors that contribute to important pathways associated with major depression: Diet, sleep and exercise. J. Affect. Disord..

[52] Klaperski S., Von Dawans B., Heinrichs M., Fuchs R. (2014). Effects of a 12-week endurance training program on the physiological response to psychosocial stress in men: A randomized controlled trial. J. Behav. Med..

[53] Moylan S., Eyre H., Maes M., Baune B., Jacka F., Berk M. (2013). Exercising the worry away: How inflammation, oxidative and nitrogen stress mediates the beneficial effect of physical activity on anxiety disorder symptoms and behaviours. Neurosci. Biobehav. Rev..

[54] Scheef L., Jankowski J., Daamen M., Weyer G., Klingenberg M., Renner J., Mueckter S., Schürmann B., Musshoff F., Wagner M. (2012). An fMRI study on the acute effects of exercise on pain processing in trained athletes. Pain.

[55] Sañudo B., Carrasco L., De Hoyo M., McVeigh J.G. (2012). Effects of exercise training and detraining in patients with fibromyalgia syndrome: A 3-Yr longitudinal study. Am. J. Phys. Med. Rehabil..

[56] Tran S.T., Guite J.W., Ounpuu S., Rodriguez-MacClintic J., Zemel L., Zempsky W., Kashikar-Zuck S., Pantaleao A., Pfeiffer M., Myer G.D. (2017). Preliminary outcomes of a cross-site cognitive-behavioral and neuromuscular integrative training intervention for juvenile fibromyalgia. Arthritis Rheum..

[57] Ang D.C., Kaleth A.S., Bigatti S., Mazzuca S., Saha C., Hilligoss J., Lengerich M., Bandy R. (2011). Research to encourage exercise for fibromyalgia (REEF): Use of motivational interviewing design and method. Contemp. Clin. Trials.

[58] Giannotti E., Koutsikos K., Pigatto M., Rampudda M.E., Doria A., Masiero S. (2014). Medium-/Long-term effects of a specific exercise protocol combined with patient education on spine mobility, chronic fatigue, pain, aerobic fitness and level of disability in fibromyalgia. BioMed Res. Int..

